# The Comet Cometh: Evolving Developmental Systems

**DOI:** 10.1007/s13752-015-0203-5

**Published:** 2015-02-17

**Authors:** Johannes Jaeger, Manfred Laubichler, Werner Callebaut

**Affiliations:** 1 EMBL/CRG Research Unit in Systems Biology, Centre for Genomic Regulation (CRG), Barcelona, Spain; 2 Universitat Pompeu Fabra, Barcelona, Spain; 3 Wissenschaftskolleg zu Berlin, Berlin, Germany; 4 School of Life Sciences, Arizona State University, Tempe, AZ USA; 5 Santa Fe Institute, Santa Fe, NM USA; 6 Marine Biological Laboratory, Woods Hole, MA USA; 7 Max Planck Institute for the History of Science, Berlin, Germany; 8 The KLI Institute, Klosterneuburg, Austria

**Keywords:** Dynamical systems theory, Epistemology, Evolutionary developmental biology (EvoDevo), Evolutionary systems biology, Natural history of configuration space, Scientific perspectivism

## Abstract

In a recent opinion piece, Denis Duboule has claimed that the increasing shift towards systems biology is driving evolutionary and developmental biology apart, and that a true reunification of these two disciplines within the framework of evolutionary developmental biology (EvoDevo) may easily take another 100 years. He identifies methodological, epistemological, and social differences as causes for this supposed separation. Our article provides a contrasting view. We argue that Duboule’s prediction is based on a one-sided understanding of systems biology as a science that is only interested in functional, not evolutionary, aspects of biological processes. Instead, we propose a research program for an evolutionary systems biology, which is based on local exploration of the configuration space in evolving developmental systems. We call this approach—which is based on reverse engineering, simulation, and mathematical analysis—the natural history of configuration space. We discuss a number of illustrative examples that demonstrate the past success of local exploration, as opposed to global mapping, in different biological contexts. We argue that this pragmatic mode of inquiry can be extended and applied to the mathematical analysis of the developmental repertoire and evolutionary potential of evolving developmental mechanisms and that evolutionary systems biology so conceived provides a pragmatic epistemological framework for the EvoDevo synthesis.

## Introduction

The field of evolutionary developmental biology, or EvoDevo, is experiencing a conceptual crisis on many fronts. One issue is that it is not a unified discipline and its limits are hard to define. Duboule—in a paper entitled “The evo-devo comet”—calls evolutionary developmental biology a “portfolio of concepts,” extending “from simply ‘PCRing’ a trendy gene from a weird animal, up to the most sophisticated molecular genetic approaches dealing with the evolution of gene function and regulation” (2010, p. 489). We largely agree with this pointed diagnosis, but argue that the current lack of conceptual unification is not an overly serious problem that we need to lose much sleep over; rather it is a sign of conceptual and theoretical development. More worrisome, in our opinion, are the following three points of conceptual deadlock.

First, traditional comparative approaches to the evolution of development—whether focused on the morphological or on the molecular/genetic level—are reaching their limits in terms of explanatory power. The more we learn about the evolution of pattern-forming gene networks, or the ontogeny of complex morphological traits, the more it becomes clear that it is less than straightforward to conclude anything about evolutionary origins or dynamics based on such comparisons alone. On the one hand, homoplasy or convergent evolution abounds at all levels of investigation. One of the most lauded major insights of EvoDevo is that a common toolkit of genes and signaling pathways is reused over and over again to create a large diversity of different body plans, shapes, and organs (see, for example, Holland 1999; Carroll 2008; De Robertis 2008). Because of this, similarities in gene expression patterns or morphological structure often do not necessarily imply common ancestry, since they may as well reflect the frequent reuse of the same regulatory or morphogenetic modules. On the other hand, developmental system drift allows conserved networks to change considerably in terms of their component genes and regulatory interactions without changing the phenotypic outcomes such systems produce (Weiss and Fullerton 2000; True and Haag 2001; Weiss 2005; Haag 2007; Pavlicev and Wagner 2012). This means that even functionally conserved regulatory networks can become unrecognizably divergent at the molecular and genetic level, especially across large evolutionary time spans. Complications such as homoplasy and system drift are most serious if the comparison is made—as it often is—between relatively distant taxa. A more fine-grained, mechanistic, causal understanding of developmental and evolutionary dynamics will be required to overcome these limitations.

The second deadlock concerns the integration of ecology or, more precisely, the active role of the environment in phenotypic evolution. Over the last few decades, it has become increasingly clear that genes and genetic programs are simply not sufficient to explain the ontogeny of most morphological traits (see, for example, Goodwin 1982; Oster and Alberch 1982; Nijhout 1990; Alberch 1991; Webster and Goodwin 1996; Keller 2000; Pigliucci 2010). Instead, a more interactive view has emerged—treating genes and their organismic as well as external environment as influencing each other in a regulative feedback loop (e.g., Waddington 1957; West-Eberhard 1998, 2003; Odling-Smee et al. 2003; Kirschner and Gerhart 2005; Gerhart and Kirschner 2007; Gilbert and Epel 2009; Moczek 2012). In this view, the environment is not just passively endured by an organism, determining its chances of survival. It plays an active and essential role in development through phenotypic plasticity (West-Eberhard 2003; Gilbert and Epel 2009), and is itself altered by the activity of the organism (Odling-Smee et al. 2003). An obvious example of the latter is humanity’s ability to massively change and manipulate the environment to our own (short-term) liking and comfort. A number of useful concepts, such as facilitated evolution (Kirschner and Gerhart 2005; Gerhart and Kirschner 2007), genetic accommodation (West-Eberhard 1998, 2003, 2005a, b), and niche construction (Odling-Smee 1995; Laland et al. 1999; Odling-Smee et al. 2003) have been proposed to tackle this challenge, but a unifying and rigorous framework to deal with the active role of the environment in developmental evolution is still missing (Moczek 2012).

The third and last deadlock concerns our difficulty in connecting (macro-)evolutionary comparisons of developmental processes to evolutionary dynamics at the population and species level. In this case, the main challenge is to find suitable model systems that allow us to combine detailed studies of the mechanisms of development with accessible and informative measures of phenotypic trait variation between closely related species or, even better, between individuals within particular populations. One of the main questions in this area is whether developmental processes evolve through mutations of small effect, affecting many loci, or whether mutations of central regulator genes with more drastic consequences play any role at all (see, for example, Akam 1998; Orr 2005; Stern 2010). There is much promising progress in this field (recently reviewed in Nunes et al. 2013). However, we only understand very few developmental processes in a small number of model organisms in a detailed mechanistic way. For this reason, most population-oriented EvoDevo studies still rely on statistical-correlative rather than on causal models of genetic architecture.

In the view of two of us (JJ and ML), all of these deadlocks could be overcome by rigorous investigations of genotype-phenotype maps in specific, experimentally tractable, developmental systems (Alberch 1991; Wagner and Altenberg 1996; Pigliucci 2010; Davidson 2011, 2014; Peter and Davidson 2011; Félix 2012). This can be achieved by the emerging methodology and conceptual framework of evolutionary systems biology (a range of perspectives on this emerging field of research are presented in Soyer 2012; see also Soyer and O’Malley 2013). What we need is a predictive model of the genotype-phenotype map that accurately incorporates the influence of both genes and the environment (see also Moczek 2012). Duboule agrees with this point, stating that the conceptual gap between developmental and evolutionary biology can be bridged “once the mechanisms of development are fully understood and once the computation of various ontogenetic roadmaps will discriminate the possible from the impossible, thus telling us which form could evolve out of a given species” (2010, p. 489). Rather pessimistically though, he argues that the current shift of developmental biology towards systems approaches impedes, rather than helps, the reunification of evo and devo, and that such a reunification may well take another century to complete.

Here, we would like to present a more optimistic outlook. We suspect that Duboule’s pessimism may at least partially be caused by his underestimating the potential of evolutionary systems biology and the rapid pace with which computational and synthetic approaches to the evolutionary analysis of complex regulatory systems are becoming a reality (see, for example, Erwin and Davidson 2009; Davidson 2011; Peter and Davidson 2011; Soyer 2012; Soyer and O’Malley 2013). One particularly promising flavor of this approach is the theory of evolving dynamical systems (see, for example, Goodwin 1982; Oster and Alberch 1982; Webster and Goodwin 1996; François and Siggia 2012). Dynamical systems theory (Strogatz 2000; Hirsch et al. 2004) provides a powerful conceptual framework that not only enables us to break the deadlocks described above, but also allows us to unify many of the currently divergent approaches to EvoDevo. One of us has reviewed some dynamical systems concepts and their potential applications elsewhere (Jaeger and Crombach 2012; Jaeger et al. 2012a; Jaeger and Monk 2014; Jaeger and Sharpe 2014). Here, we will only briefly reiterate some of the main points relevant to our current discussion.

## Epistemological Foundations: It’s a Matter of Perspective

Before we can define our proposed new research program for evolutionary systems biology more precisely and put it into historical context, we need to clarify an important philosophical aspect of our argument. As we have mentioned above, one of the main points of criticism raised against EvoDevo—or, more generally, against the idea of an extended evolutionary synthesis—is that there is no consistent compact conceptual framework to unify development, ecology, and evolution (as stated in no uncertain terms in Duboule 2010). Instead, the proposed Extended Synthesis includes a great diversity of experimental and conceptual approaches, all focused around seemingly disparate questions and problems such as developmental constraints, epistasis, robustness, evolvability, modularity, non-genetic inheritance, phenotypic plasticity, and niche construction (see above, and Müller 2007; Pigliucci 2007; Pigliucci and Müller 2010; Moczek 2012; Laland et al. 2014).

Within this diversity of approaches, some prominent researchers in the field address this problem of methodological and conceptual diversity by restricting the scope of EvoDevo to molecular mechanisms of regulatory evolution (e.g., Holland 1999; Carroll et al. 2004; Duboule 2010). In this view, EvoDevo is rooted in the experimental tradition of developmental genetics, and its origins can be traced back to the discovery of the Hox genes in the early 1980s (this revisionist view of history was first explicitly presented in Holland 1999). However, there are older branches of the field, which put their focus on the morphological level. These are the traditions of comparative embryology and morphology (including extant and fossil specimens), centered around the concept of morphological homology, and what has been called experimental epigenetics, the study of morphogenetic aspects that are not controlled directly by genetic factors (see, for example, Goldschmidt 1940; Waddington 1957; Gould 1977; Gould and Lewontin 1979; Hall 1999; Amundson 2005; Müller and Newman 2005; Laubichler and Maienschein 2007; Müller 2007; Wagner 2014). A third current in EvoDevo is the study of the genetic variation underlying phenotypic differences within populations or between species (reviewed in Nunes et al. 2013; Stern 2010). This type of research, with its focus on evolutionary rather than developmental dynamics, is much more closely related to evolutionary genetics than the two other approaches. It uses statistical genotype-to-phenotype associations in variable populations, rather than mechanistic comparisons of developmental processes, as its main approach. Finally, there is the small but vigorous tradition of computational EvoDevo, which blends into the wider computational fields of in silico evolution and artificial life (e.g., Goodwin 1982; Oster and Alberch 1982; Newman and Comper 1990; Alberch 1991; Newman 1994, 2006, 2012; Salazar-Ciudad et al. 2000, 2001a, b; Salazar-Ciudad and Jernvall 2002, 2004, 2010; François et al. 2007; Fujimoto et al. 2008; Nahmad et al. 2008; Wagner 2008, 2011; Newman and Bhat 2009; Draghi et al. 2010; François and Siggia 2010, 2012; Hoyos et al. 2011; ten Tusscher and Hogeweg 2011; Jaeger and Crombach 2012; Peter et al. 2012; Salazar-Ciudad and Marín-Riera 2013; Crombach et al. 2014; Jaeger and Sharpe 2014).

This diversity within EvoDevo sometimes impedes communication among practitioners in the field, thus posing a practical problem. However, we argue that it is not really an issue from a theoretical, epistemological point of view. On the contrary, this diversity of approaches and questions is positively required to tackle the problems of EvoDevo, exactly because these problems are extremely diverse themselves. The evolution of development can be studied in many contexts, in different organisms, considering different stages of development, organs, tissues, or other cellular contexts, with many different aspects of the process in mind. In fact, the complex and historically contingent (and hence messy) nature of developmental evolution makes it absolutely essential to take a pluralistic, pragmatic approach. There is an almost inexhaustible number of possible questions and many possible levels of explanation that complement and inform each other, even though they may never be integrated into a grand unified general theory of evolving developmental systems.

Different explanations can focus on different scales (molecular, morphological, organismic, or even ecological), or can be framed in different conceptual frameworks. We can try to understand evolution in terms of gene conservation, for example, or the conservation of regulatory network structure. The former provides explanations in terms of the constituents of a network (a molecular parts list, with an associated historical trajectory of these parts). The latter requires much different concepts to study and understand the developmental repertoire and evolutionary potential of the system (see below). Such concepts describe the interrelations or biological organization of interactions between network parts. These alternatives are not at all mutually exclusive, but neither do they seamlessly overlap. Instead, they complement each other and enrich our explanatory toolkit by adding new perspectives on a common underlying theme. It is as if we are looking at the problems of EvoDevo from different angles, and while these views yield an increasingly integrated and powerful understanding of the subject of our study, they are unlikely to result in a “God’s eye” view: a simple and universal theory of developmental evolution that can be formulated on the back of an envelope.

Such a diversified approach is justified by the philosophy of scientific perspectivism (Giere 2006; Wimsatt 2007; Van Fraassen 2008; Callebaut 2012). Its basic tenet can be summarized as follows. Perspectivism, as formulated by Giere (2006) or Wimsatt (2007), views science as a process occurring between particular scientists and the causal structure of reality. On the one hand, it is a form of realism, acknowledging a physical universe independent of the observer. On the other, perspectivism also considers the human subjective aspect of the scientific endeavor: scientists build models of specific processes with a given purpose in mind. This purpose depends on the background and motivation of the individual researcher, as well as the social context of the scientific community. Different scientists want answers to different questions, explanations at different levels (see also the accompanying article by Green et al. 2014, this issue). Some explanations that were useful fifty years ago are no longer considered so today. However, scientific progress is neither linear nor simple. New explanations do not simply replace older ones, leading to an increasingly accurate picture of reality. Instead, each explanation provides a unique perspective on reality. Different perspectives can complement each other, and should be consistent in areas where they overlap, but they will never add up to a complete or objective theory of the physical world. In other words, there is no theory of everything. Instead, perspectivist theories correspond to local models that address a specific problem within a given scope at a given time.

In this pragmatic spirit, we propose that a dynamical-systems view of evolving developmental processes provides a new, powerful perspective on EvoDevo, a new angle on the central questions in the field. This perspective can help us transcend the limitations of traditional comparative approaches at the morphological or molecular level. It also neatly complements the approach of mapping genetic variation of developmental traits at the population level. In this sense, it neither contradicts nor diminishes the merit of other branches of EvoDevo, but rather extends them by defining the evolutionary potential of developmental systems.

## The Promise of Evolutionary Systems Biology

Dynamical systems theory has a long and successful history of investigating cellular and developmental regulatory processes (see, for example, Turing 1952; Britten and Davidson 1969; Kauffman 1969, 1993; Thom 1976; Meinhardt 1982; Webster and Goodwin 1996; Davidson 2001, 2006; Murray 2002; Kaneko 2006; Huang 2009, 2012; Jaeger 2009; Kondo and Miura 2010; Hogeweg 2011; Wagner 2011; Jaeger et al. 2012b; Oates et al. 2012). Today, interest in systems-biology approaches for cell and developmental biology is higher than ever, as evidenced by several recent focus issues and special sections on the topic in various journals (e.g., *Science*, 2012, vol. 338, pp. 209–219; *Current Opinion in Genetics & Development*, 2012, vol. 22, pp. 523–633; *The* *Journal of Physiology*, 2014, vol. 592, pp. 2237–2438). In contrast, the application of dynamical systems theory to EvoDevo remains rare and somewhat marginal. Early attempts focused on conceptual issues and analysis but lacked data (e.g., Waddington 1957; Thom 1976; Goodwin 1982; Oster and Alberch 1982; Newman and Comper 1990; Alberch 1991; Goodwin et al. 1993; Kauffman 1993; Newman 1994; Webster and Goodwin 1996). Recent modeling work is more closely connected with empirical evidence, but has lost some of its conceptual focus since it is mainly based on numerical simulation methods without much of an analytical component (e.g., Salazar-Ciudad and Jernvall 2002, 2004, 2010; Nahmad et al. 2008; Hoyos et al. 2011; Peter et al. 2012; Salazar-Ciudad and Marín-Riera 2013).

We propose to combine the conceptual and empirical aspects of these previous studies for a new kind of systems-level EvoDevo. Our integrative approach combines quantitative data, modeling, and dynamical systems analysis to reverse engineer a large variety of different developmental systems and their underlying regulatory mechanisms (Jaeger and Crombach 2012; Jaeger and Sharpe 2014). The overarching aim is to derive generalizable principles of evolutionary and developmental dynamics from a functional classification of such a collection of developmental mechanisms (as discussed in Jaeger and Sharpe 2014, and the accompanying paper by Green et al. 2014). This constitutes one possible way of defining a unified research program for evolutionary systems biology (see Soyer and O’Malley 2013 for further discussion).

Dynamical systems theory investigates biological regulatory processes by formulating, simulating, and analyzing mathematical or computational models that represent the underlying complex non-linear dynamics (Strogatz 2000; Hirsch et al. 2004). These models are based on equations or algorithms (de Jong 2002; Karlebach and Shamir 2008) that encode the set of rules—or the regulatory structure—governing a system’s repertoire of dynamic behaviors (Alon 2006; Bolouri 2008). Even for complex, non-linear systems, it is possible to analyze this dynamical repertoire in a qualitative way (Strogatz 2000; Neuenschwander 2013): alternative pattern-forming or phenotypic outputs of a process are defined by a number of attractor states, with their associated basins of attraction (Jaeger and Crombach 2012; Jaeger et al. 2012; Jaeger and Monk 2014; Jaeger and Sharpe 2014). As an example, let us look at a bistable toggle switch model, which is represented by a small network of two mutually repressing regulatory genes. The state variables of the system are defined by the concentrations of regulator gene products. This toggle switch exhibits two alternative output states in which either of its two components is switched on, while the other one is shut off. These alternative states, with their characteristic regulator concentrations, correspond to the two attractors of the system (see Jaeger et al. 2012, and references therein). Model parameters represent production and decay rates, or the type and strength of regulatory interactions in the network. Genes and environmental influences jointly determine the initial state and the parameter values of the system. During the process of development, state variables change, and parameters can be altered due to internal or external signals or triggers (Verd et al. 2014). During evolution, the initial state and the parameter values are varied through mutations and seasonal or permanent changes in the environment (Jaeger et al. 2012; Jaeger and Monk 2014).

State variables and system parameters together define the axes of an abstract space called configuration space (Thom 1976; Jaeger and Sharpe 2014). Development and evolution propel the system through this space. On its journey, it encounters boundaries at which its behavior switches to an alternative attractor, or where attractor states are created or annihilated through the process of bifurcation (Strogatz 2000; Hirsch et al. 2004; Kuznetsov 2004). We will not go into detail here, but the main point to keep in mind is the following: if we can characterize the geometry of configuration space for a specific evolving developmental process—that is, the arrangement of its attractors, their basins, and their bifurcations—we can predict which phenotypic transitions can or cannot occur, and how probable particular phenotypic changes will be, given some sort of selective pressure or neutral drift. In other words, knowing the configuration space of a system, we can define its potential for evolutionary innovation and change. This problem—the problem of evolvability—is a central challenge, not only for EvoDevo (Hendrikse et al. 2007). It also forms one of the central pillars of a proposed Extended Synthesis for evolutionary biology that includes EvoDevo as one of its main components (Müller 2007; Pigliucci 2007; Pigliucci and Müller 2010; Laland et al. 2014). Dynamical systems theory harbors the promise of enabling us to tackle this important issue at the very core of modern biology.

A critical reader may object at this point that dynamical systems theory has been harboring this promise for a while now without delivering any specific results. We agree to some extent. Our argument in the preceding paragraphs is not new, but has been made previously in various forms by other authors (Thom 1976; Goodwin 1982; Oster and Alberch 1982; Newman and Comper 1990; Alberch 1991; Goodwin et al. 1993; Kauffman 1993; Newman 1994, 2012; Webster and Goodwin 1996; François and Siggia 2012; Huang 2012). What *is* new is that—for the first time in history—we can combine such theoretical approaches with quantitative empirical evidence, using the data sets and methodologies generated by modern systems biology (Jaeger and Crombach 2012; Jaeger and Sharpe 2014). This allows us to reverse engineer the regulatory networks underlying specific cellular and developmental processes, and to test our models and concepts against detailed and accurate measurements of gene expression pattern or morphological trait characteristics. This opens up exciting novel avenues for research—a new kind of evolutionary systems biology—that may completely transform the fields of EvoDevo and evolutionary biology, and turn them from purely historical into locally predictive branches of science.

## A Pragmatic Approach: Natural History, Not Global Mapping

One of the main reasons for Duboule’s (2010) pessimism about the return of the EvoDevo comet is the staggering complexity and diversity of cellular and developmental regulatory processes. The configuration space for realistic models of such systems is vast, high dimensional, and potentially infinitely complex. For this reason, it may be a bit too optimistic to try and formulate a general theory of its geometry at this point. In fact, such an overarching theory has been proposed by René Thom (1976) who proved that the number of different possible morphogenetic processes is surprisingly small if considering only a limited, precisely defined, class of dynamical systems. This rigorous analytical insight is intriguing and encouraging. Unfortunately, however, most real-world regulatory networks do not fall into the limited class of systems to which Thom’s proofs apply.

We would like to contrast this approach, which we may call global mapping of configuration space since it charts the geometry of all possible regulatory systems, with a more modest, pragmatic one. Following our perspectivist outlook, we are not looking for general theories but for a more local perspective. We propose something resembling a series of targeted expeditions into the vast, unknown territory of configuration space. Although we cannot gain a complete overview of the landscape yet, surely we could gain a lot of exciting new insights by just having a look around! This seems a sensible approach in light of the fact that nobody has ever explored this territory before. We call this *the natural history of configuration space*, in analogy to the real-world expeditions that have led humanity to discover new countries, continents, and even new worlds beyond our own planet. Such expeditions always preceded the systematic mapping of the newfound frontier, not only in natural history proper, but also in many other branches of biology and science. Much of the evidence in evolutionary biology and, more specifically, in EvoDevo with its focus on a limited number of model systems, remains of a contingent and often somewhat anecdotal nature. In an analogous vein, simulation studies of evolving networks do not attempt to develop a general theory, but simply explore plausible evolutionary scenarios. These very successful pragmatic strategies are similar to our suggested exploration of configuration space in that they provide targeted local insights. Therefore, our proposal is very much in line with the history and philosophy of evolutionary theory and EvoDevo so far.

In the following sections, we will illustrate this idea using examples from three different areas. We first review the role that natural history has played in the development of evolutionary theory. We then discuss the traditional approach of using experimentally tractable model systems as exemplars of “typical” developmental processes in EvoDevo. We proceed to provide a brief overview of simulation-based studies of network evolution. Similar to experimental studies in model systems, these simulations represent a small but informative sample for what kind of behaviors could be expected from regulatory networks in general. Finally, we illustrate our proposed pragmatic approach to the study of the configuration space of developmental systems. Judging by past successes within the former three areas, this approach bears great promise for providing us with—necessarily biased and incomplete—but nevertheless useful and locally generalizable insights into the functional and evolutionary potential of developmental systems.

### The Story So Far: Natural History and Evolutionary Theory

The history of biology provides us with some interesting perspectives on the role theory has played within the life sciences (the current term for what has been a very diverse set of approaches and disciplines over the last 2,500 years). One of the most persistent problems has been the question whether living systems need to be explained by their own set of guiding principles or whether they are simply a (complex) product of underlying physicochemical interactions. One can write the whole history of biology as a sequence of different ways of explaining the nature or essence of living systems, starting with Aristotle’s notion of four causes all the way to the molecular conception of life that emerged in the middle of the 20th century. In this context, systems biology is one of the latest attempts to identify exactly what kind of interactions are necessary in order to understand living systems.

Another approach to explaining the unique features of living organisms has focused on their evolutionary history. Many biologists agree with Dobzhansky’s famous dictum that “nothing in biology makes sense, except when seen in the light of evolution” (Dobzhansky 1973). This statement is generally thought to imply that evolutionary theory is the only genuine theory of biology. While we do not want to enter this (somewhat academic) discussion (see Krakauer et al. 2011 for a more nuanced view), we certainly agree that evolutionary theory is indeed a foundational theoretical framework for understanding living systems. The historical origins of evolutionary theory are therefore especially informative for our reflections about the epistemological status of EvoDevo and evolutionary systems biology.

Historians have reconstructed the emergence of evolutionary theory in great detail. What is most relevant for our argument is the fact that evolutionary theory grew out of the tradition of natural history, which focused on systematically studying and documenting the diversity of life. An original emphasis on diversity has thus eventually given rise to a unifying theoretical framework. Now, when Duboule (2010) and others argue that EvoDevo lacks unified principles and is mainly a collection of different approaches, model organisms, experimental systems, and their associated concepts, we see this not as a problem, but rather as a necessary step in the historical sequence leading to a more general theoretical framework.

In this context we also argue, and so do many of our colleagues, that evolutionary theory itself is still evolving, as is evidenced by the recent debate over the adequacy of standard evolutionary theory (Laland et al. 2014). This controversy is mainly about the best ways to integrate recent empirical and theoretical advances within evolutionary biology and related fields with the core assumptions of evolutionary theory. Among the advances in need for integration are insights from molecular and developmental biology that have led to the concepts of developmental and regulatory evolution and genomic regulatory networks (Davidson 2001, 2006, 2009, 2011; Materna and Davidson 2007; Carroll 2008; Shubin 2008; Stern and Orgogozo 2008, 2009; Krakauer et al. 2011; Peter and Davidson 2011; Peter et al. 2012; Ben-Tabou De-Leon et al. 2013), and a deeper integration of ecological and evolutionary theory that has refocused attention on complex phenomena such as phenotypic plasticity (West-Eberhard 2003; Moczek 2012) or the idea of niche construction with its focus on multiple inheritance systems (Odling-Smee 1995; Laland et al. 1999, 2008; Odling-Smee et al. 2003; Laland and Sterelny 2006; Jeffares 2012; Odling-Smee et al. 2013; Richerson and Christiansen 2013; Buser et al. 2014). However, in all these cases the question has been whether new data and concepts or new explanatory domains can be accommodated within the existing framework of evolutionary theory, or whether the core of evolutionary theory needs to be reconceptualized or, at the very least, expanded. One of the central questions in this context is the distinction between the origin of variation and the fate of variants once they exist within populations.

The focus on regulatory networks, such as gene regulatory networks, helped to discover causal mechanisms that control the development of specific phenotypic characters. Furthermore, comparative studies (of different species and of normal and pathological conditions) have shown how specific transformations of either regulatory network structures or individual elements within those networks are responsible for observed phenotypic variation (Carroll 2000, 2008; Wagner et al. 2000; Davidson 2006, 2014; Peter and Davidson 2011; Wagner 2014). While many of these studies have focused on the genome, it has, however, also become clear that many contextual factors interact with the genome-based control circuits and thus contribute to the regulation of gene expression in a significant way (Linksvayer et al. 2011, 2012; Page 2013). The specific nature of these interactions can, in principle, be traced outward from the genome and involves intra- and extracellular signaling pathways, metabolic and physiological networks, behavior, and specific environmental factors that can all contribute to such regulatory cascades. In practice, however, detailed reconstructions of such extended causal networks are still rare and specific contextual effects are generally subsumed under a generalized environmental contribution to the partition of variance, and in any case are considered to be a factor that is independent from the genomic, cellular, or organismal system.

Niche construction theory, on the other hand, focuses on the ways systems actively shape or construct their environment. In this view, the niche is not something that exists out there in nature waiting to be discovered or filled by an organism. Furthermore, constructed niches often persist longer than any of their individual inhabitants, which allow these niches to store important hereditary and regulatory information. Niche construction theory thus includes the notion of expanded and multiple inheritance systems (from genomic to ecological, social, and cultural). This latter aspect has made the concept of niche construction especially attractive for theories of cultural evolution as it facilitates a more complex notion of inheritance and a closer link between evolutionary dynamics and learning (Odling-Smee 1995; Laland et al. 1999, 2000; Boyd and Richerson 2005; Laland 2008; Laland et al. 2008; Boyd et al. 2011; Creanza et al. 2012). But most models of niche construction have treated these multiple inheritance systems as quasi-independent contributions to evolutionarily relevant variation, allowing only limited interactions between them. In part this is a consequence of the formal structure of variance decompositions (the famed Price equation) that is the foundation of much of niche construction theory. But it also reflects a tendency within niche construction theory to focus on multiple broadly defined factors and quantify their relative importance within evolutionary dynamics.

What both of these approaches are missing is a clearly defined conception of how systems at multiple scales interact with each other, where some are defined as internal to the organizational level of study and some are defined as context or environment. A precise definition of the nature of these interactions is, however, a prerequisite for a causal model of the evolution of complex systems and also for understanding innovation across scales. This requires us to clearly define the relevant elements of these systems and their properties. Without conceptual precision it will be impossible to define the measurements and metrics needed to turn integrative conceptual ideas into formal models and to specify the criteria for empirical validation. Another challenge is to trace the consequences of causal interactions at different scales through an iterative sequence of historical stages.

EvoDevo, developmental evolution, and evolutionary systems biology are all attempts to conceptualize these transformations of evolutionary theory. All of them rely to large degrees on multiple models and experimental systems; they are, in analogy to the origin of evolutionary theory, all transitioning from a natural history phase to an integrated theory phase.

### The Digital Revolution: The Natural History of Computational Models

The importance of “natural history”—local exploration as opposed to global mapping—is obvious in the context of evolutionary theory and experimental EvoDevo. Less obviously, we argue that simulation-based studies of evolving regulatory networks and developmental processes also conform to a natural-history-like mode of inquiry. To illustrate this, we use a number of selected examples from three subdisciplines representing alternative approaches to the computational study of regulatory network evolution (Jaeger and Crombach 2012): ensemble simulations, in silico evolution, and detailed data-driven modeling of specific evolving developmental systems.

The ensemble approach simulates large sets of networks that belong to a given class (Kauffman 2004): members of a network ensemble differ in their specific regulatory structure but share certain other characteristics. The aim is to discover whether there are network-level features that depend only on those common characteristics, not on the fine-grained details of regulatory wiring.

The ensemble approach was pioneered by Stuart Kauffman. Working with simple Boolean logical models of large regulatory networks, Kauffman discovered that the number of attractors (and hence phenotypic outcomes) in a system is always small compared to the total number of components (Kauffman 1969). Furthermore, networks that process inputs through a peculiar type of regulatory function—so-called canalizing functions whose output only depends on a single control input—were found to be more robust to perturbation than others (Kauffman 1974). More recently, ensemble simulations have given us interesting and important new insights into the connection between robustness and evolvability. Simulations of networks that share a scale-free overall connectivity, for instance, reveal that they are more robust towards parameter perturbations than a control set of randomly wired systems (Aldana 2003; Aldana and Cluzel 2003). And yet, these networks are more evolvable, since they tend to preserve attractor states upon duplication of network nodes (Aldana et al. 2007). Robustness of patterning in general relies on the simple observation that there are many more possible network structures (called “genotypes” in the context of gene regulatory networks) than output patterns (“phenotypes”) (Kauffman 1993; Borenstein and Krakauer 2008; Munteanu and Solé 2008). In other words, many different regulatory structures produce the same output. Interestingly, many of these structures are connected through single mutational steps: it is usually possible to change at least one regulatory interaction in a network without altering the phenotype it produces. This results in large connected sets of regulatory structures called “genotype networks” (Ciliberti et al. 2007a, b; Wagner 2008, 2011; Draghi et al. 2010). Genotype networks can span a substantial proportion of the space of all possible network structures, enabling evolving systems to drift across large proportions of genotype space while maintaining their phenotypic output. The further they drift, the more neighboring, potentially adaptive, phenotypes become accessible through mutations. This resolves the apparent conflict between robustness to mutation and evolvability, two of the central characteristics of biological regulatory networks (Wagner 2008, 2011).

Apart from the discovery and characterization of general network-level properties, the ensemble approach is also suited for the systematic exploration of different regulatory mechanisms that can implement a given biological function. A recent study by Cotterell and Sharpe (2010), for example, discovered that there are only six basic ways in which three-gene networks can produce a stripe of gene expression within a tissue in response to the graded distribution of a morphogen gradient. While some of these mechanisms had been observed in nature, others are yet to be found experimentally. In this way, a network atlas based on ensemble simulations can provide a map of the possible, the space of possibilities that evolution by natural selection can explore (Jaeger and Sharpe 2014).

While ensemble studies establish that a specific mechanism exists in principle, they do not teach us whether it can actually evolve. For this reason, the ensemble approach needs to be complemented with in silico evolution (Jaeger and Crombach 2012). In silico evolution simulates populations of mutating networks, whose output is measured against some fitness function such that networks with higher fitness will be more likely to propagate into subsequent generations.

Many in silico evolutionary simulations have been carried out in the context of body segmentation. Segmented body plans occur in diverse and successful groups of animals, such as annelids, arthropods, and vertebrates (Peel and Akam 2003; Tautz 2004; Couso 2009; Chipman 2010). Segments become determined in one of two ways during development: they either arise through growth and sequential addition at the posterior end of the embryo, or simultaneously, through subdivision of the embryo without growth. A pioneering in silico evolutionary study suggested that an oscillatory mechanism—creating timed pulses of gene expression—can also produce a periodic spatial pattern if put in a syncytial embryo (Salazar-Ciudad et al. 2001b). It revealed that sequential segmentation mechanisms tend to have a less hierarchical regulatory structure than those evolved towards simultaneous segment determination (Salazar-Ciudad et al. 2000, 2001a). Later studies corroborated and extended these results. Simultaneous segmentation is based on feed-forward regulatory motifs, such as cascades of repressors, while sequential segmentation relies on feedback-driven oscillations (François et al. 2007; Fujimoto et al. 2008). Interestingly, evolving a sequential segmentation mechanism involves a so-called “evolutionary funnel,” a specific type of developmental constraint on evolutionary trajectories: bistability always has to evolve first in such a system, before an oscillatory clock mechanism can drive the periodic appearance of stripes (François et al. 2007). Finally, segment determination and diversification of individual segments can evolve at the same time, or determination before diversification; however, regulatory networks evolved in the latter way are more robust than the former, due to increased modularity of the resulting regulatory structure (ten Tusscher and Hogeweg 2011).

In silico evolution has also been used to study evolvability. Kashtan and colleagues (Kashtan and Alon 2005; Kashtan et al. 2007) showed that modularity in network structure evolves in response to selective pressure to solve a modular problem. Rupert Riedl called this phenomenon the “imitatory epigenotype” (Riedl 1978). Another set of evolutionary simulations focused on regulatory dynamics rather than network structure. It revealed that networks evolving in a recurrently changing environment acquired so-called “evolutionary sensors,” which allow them to adapt to the alternative environment with increasing rapidity over time (Crombach and Hogeweg 2008). These sensors are particular regulatory subsystems that remodel the geometry of configuration space in a way which facilitates mutational access to both alternative patterning outputs.

At first sight, both the ensemble approach and in silico evolution seem to be top-down global mapping approaches. They aim to systematically sample configuration space, or to simulate all possible evolutionary trajectories. On closer examination, however, we realize that neither of them is truly exhaustive as only an analytical approach can be. This is due to two important limitations. First, numerical sampling can never lead to a complete description of a dynamical repertoire. Due to its coarse-grained nature, we may always be missing some important detail. Second, and more importantly, a lot of the assumptions underlying the models presented here—discretization of space and/or time, choice of modeling formalism, mutational operator, and fitness function, etc.—remain necessarily ad hoc. We know far too little about eukaryotic transcriptional regulation, mechanisms of mutation, and specific selective pressures to derive rigorous and unique choices about the level of detail and accuracy required to properly capture some evolutionary phenomenon. All we can do is simulate plausible scenarios. In other words, each ensemble or evolutionary simulation is an expedition into the vast space of possibilities available to evolution.

One last simulation-based approach is worth mentioning at this point: detailed evidence-based modeling of specific evolving developmental processes. The literature on this topic is still rather small, mainly because not many systems have been studied in this way so far. One example is the long-running effort to model mammalian tooth development and evolution by Jukka Jernvall, Isaac Salazar-Ciudad, and colleagues. These authors created a complex spatial model of pattern formation based on signaling interactions between differentially growing tissues within the tooth primordium (Salazar-Ciudad and Jernvall 2002, 2010). The model reveals that various tooth shapes—in mice versus voles, for example—can be simulated by altering a small number of control parameters. In addition, the model reproduces shape transitions in the fossil record, plus variation in tooth morphology of isolated lake seal populations. More generally, the model has been used to study the importance of feedback interactions between molecular patterning and tissue growth, as well as the effect of the complex non-linear structure of the genotype-phenotype map, on evolutionary dynamics (Salazar-Ciudad and Jernvall 2004; Salazar-Ciudad and Marín-Riera 2013).

It is immediately obvious that this approach is natural-history-like, similar to experimental EvoDevo studies of model organisms in general. Accurate modeling of developmental processes requires a substantial amount of experimental and computational work, and a lot of attention to relevant detail. Therefore, its application will always be limited to carefully chosen, experimentally tractable, model systems. Fortunately, the tooth example described above beautifully illustrates that insights from the study of a particular system need not be limited to the developmental process under consideration. Just as in the case of evolutionary theory and experimental EvoDevo, case studies can lead to valuable and generalizable insights without having to provide a grand unified theory of developmental evolution.

The examples we have discussed in this section illustrate the explanatory power of simulation-based approaches. They enable us to explore and understand network-level properties of evolving developmental systems, such as robustness and evolvability. They allow us to systematically map the space of the possible. They can show us what kind of evolutionary transitions are likely or unlikely to occur. However, simulation studies also have their limitations. They sample the developmental and evolutionary dynamics of regulatory networks, but they do not provide any explanations as to *why* these systems are robust, or *why* some transitions occur more frequently than others. If we want answers to these deeper questions, if we want to go below the surface of numerical simulation, we need to explore the configuration space of the dynamical systems implemented by these networks.

### A New Frontier: The Natural History of Configuration Space

Investigating the geometry of configuration space enables us to understand the developmental repertoire and the evolutionary potential of a regulatory system. Simulation-based studies explore plausible evolutionary scenarios in a natural-history kind of way. If we combine these two complementary modes of inquiry, we end up with a powerful new approach to the study of evolving networks (Jaeger and Crombach 2012; Jaeger and Sharpe 2014). The first step is to develop models of specific developmental processes, through forward modeling as described in the previous section, or through reverse engineering of dynamical systems from quantitative data. The second step simulates the range of possible evolutionary transitions between such empirically established starting and end points (in silico evolution). During the third step, the resulting models are analyzed mathematically to reveal those features of configuration space that provide causal explanations for pattern-forming and evolutionary transitions. Empirical models and evolutionary simulations provide an anchor point, a base camp so to speak, for targeted local numerical “expeditions” into configuration space. Embarking on many such expeditions is the basic idea behind our natural-history approach to configuration space analysis.

It is important to note that only the last step of this research strategy is peculiar to our proposal. In contrast, the general argument that the analysis of configuration space is essential for understanding biological regulatory systems is not new, although it has always remained marginal. Explicit connections between dynamical systems theory and what is nowadays called EvoDevo were already made by René Thom (1976). His work was based on Waddington’s epigenetic landscape (1957), a metaphor for the geometry of configuration space (Huang 2012; Jaeger and Monk 2014; Verd et al. 2014). Later, Alberch and Oster (Oster and Alberch 1982) as well as Goodwin and colleagues (Goodwin 1982; Goodwin et al. 1993; Webster and Goodwin 1996) argued that dynamical systems concepts are required to understand the interplay of development and evolution. For instance, phenotypic transitions can be understood as discrete bifurcation events (Oster and Alberch 1982), large basins of attraction explain robustness of regulatory systems (Goodwin et al. 1993), and morphogenetic fields are defined by sets of features of configuration space for spatially distributed systems (Goodwin 1982; Webster and Goodwin 1996). We review these ideas in more detail elsewhere (Jaeger et al. 2012; Jaeger and Monk 2014).

This early work was entirely theoretical. Here, we illustrate the power of configuration space analysis through the discussion of two more recent studies that are firmly based on experimental evidence. Both of them focus on developmental dynamics and the analysis of pattern-forming mechanisms. We show further below how they can be expanded to include evolution.

The first study, by Manu et al. (2009a, b), examines pattern formation during segment determination in the fruit fly *Drosophila melanogaster.* In particular, it explains features of gap gene expression—such as robustness and precision of domain boundary placement—in terms of attractors and their basins. In summary, the analysis shows that expression boundaries in the anterior of the embryo, which remain stationary over time, are established by attractors. Just as in the example of the toggle switch, some nuclei along the axis of the embryo fall into one basin of attraction, while neighboring nuclei reach another attractor with a different set of gene product concentrations. Expression boundaries in the posterior, which shift over time, are formed by a completely different kind of mechanism. They fall into an attracting structure of configuration space called an unstable manifold. This manifold induces each nucleus to cycle through a succession of gene expression states, similar to a traffic light switching its state from green to yellow to red. If put into a spatial context, this temporal switching of gene expression produces coherent spatial movement of domain boundaries (see Jaeger and Crombach 2012 for a more detailed non-technical review).

The second study, by Corson and Siggia (2012), examines the specification of vulval fate in the embryo of the roundworm *Caenorhabditis elegans.* In this case, the authors took a somewhat unusual approach to modeling. Instead of formulating a mathematical model by deriving a set of equations based on molecular evidence about gene regulation, they designed the system such that its configuration space conformed to experimental observations upon perturbation of the system. Manipulation of the so-called anchor cell, which serves as a signaling center, leads to particular patterns of reassigned cell fate in its neighbors. The model successfully predicts the frequency and probability of cell fate changes upon a number of experimental interventions. It explains commonalities and differences between the induced phenotypes through changes in configuration space geometry.

Neither of these examples addresses evolutionary aspects of the systems under study, but it is easy to see how their methodology can be adapted to such problems. One way is through comparative analysis using dynamical systems models across different organisms. Just as we can compare homologous gene expression patterns, we can compare equivalent structures in configuration space between species. Those regulatory mechanisms that have changed, and those that have remained the same across evolutionary time spans, are identified by differences and similarities in the geometry of phase space. We cannot yet illustrate the usefulness of this approach for EvoDevo by concrete examples. No such comparison of configuration spaces has yet been carried out. But it is easy to see its potential. In fact, one of us is currently carrying out such a network-level comparison of the gap gene system in different species of flies (Jaeger and Crombach 2012). Initial results are encouraging (Crombach et al. 2014).

Another way to apply configuration space analysis to evolutionary problems is to use existing data-driven models as the starting point for in silico evolutionary simulations. The advantage of this approach is that it allows intermediate transient configuration space geometries to be reconstructed and characterized, such that specific bifurcation events can be assigned to particular evolutionary transitions. Two of the current authors (JJ and ML) are attempting this sort of analysis in the context of fly segmentation, and the network governing the subdivision of the embryo into separate germ layers during early development of sea urchins, respectively.

We are very optimistic that comparative studies of configuration space will yield explanations for pattern-forming and phenotypic transitions in evolving developmental systems in the very near future. What are the types of bifurcations that drive such processes? What kind of geometrical arrangements of attractor basins are frequent? What others are rare? Which transitions cannot occur at all since the basins of attraction for their phenotypes are isolated from each other in configuration space? Can we classify the possible types of transitions in realistic models of developmental processes, very much in the spirit of Thom? These and other questions are rapidly becoming tractable, as integrative methods to reverse engineer, simulate, and analyze evolving developmental systems become more powerful and less labor intensive.

## Conclusion

In light of the examples presented here, we have to revisit Duboule’s (2010, p. 489) pessimistic forecast that the EvoDevo comet “may indeed take another century” to return, and his claims that systems biology is driving developmental and evolutionary biology apart, instead of uniting them. On the one hand, we do agree with Duboule that a truly unified and comprehensive theory of development is far beyond our current reach, and may not be possible (or even desirable) to achieve (depending on one’s meaning of comprehensive theory). On the other hand, we have argued here that evolutionary systems biology may indeed be able to provide solutions to some of the most enduring and daunting challenges in modern biology over the next few years. It will do this not by providing a deductive framework, but by offering pragmatic local models that can then be compared and classified to reveal potential regularities or rules of developmental evolution. These efforts by no means separate developmental and evolutionary developmental biology. Instead they reveal complementary perspectives: systems biology provides a map of the possible, what is likely to be encountered by natural selection, while evolutionary genetics shows how this substrate then leads to specific evolutionary dynamics. Thus conceived, evolutionary systems biology brings Darwin’s two questions—the origin and subsequent fate of variation within populations—back into focus.
